# New Appliances and Things Medical

**Published:** 1909-04-17

**Authors:** 


### NEW APPLIANCES AND THINGS MEDICAL.

[We shall be glad to receive at our Office, 23 & 29 Southampton Street, Strand, London, W.C., from the manufacturers, specimens of all new preparations and appliances.]

### TANNISMUT; COLLARGOLUM; COLLARGOL TABLETS.

(Chemische Fabrik von Heyden, Radebeul near Dresden. London Agent: E. W. Blasius, 127 Fenchurch Street, E.C.)

Tannismut is a new intestinal sedative useful for the treatment of many kinds of diarrhœa. The drug consists of bismuth combined with two molecules of tannin. It is a tasteless dust-coloured powder and can be given in doses of from 3 to 7½ grains three times a day or more often. It is also made up into tablets, each containing 0.5 gramme or 7½ grains. Clinical results have shown that it controls intestinal irritation of many kinds, and that constipation does not follow its use. Collargolum is a colloidal preparation of silver having strong antiseptic properties. It may be administered by inunction, and for this purpose an ointment termed Ung. Credé is supplied. Of this ointment 45 grains should be applied by inunction once a day. It may also be taken internally in doses of from 3 grains to 15 grains per diem. It can also be used for vaginal douches or rectal enemata, about 2 oz. of a 1 per cent. solution being required. It has been given by intravenous injection, quantities of from 1 to 4 fluid drachms of a 2 per cent. solution being used. It is supplied in tablets, each containing ¾ of a grain.

### GROVE'S COMMON-SENSE NASAL DOUCHE.

(The American Commerce Co., Ltd., 19 St. Bride Street, E.C.)

The greatly increased attention which has been paid during recent years to the local treatment of catarrhal and suppurative inflammations of the nose and its accessory sinuses has resulted in the output of numerous devices for facilitating fluid applications in this region. There are a good many nasal douches now on the market of various kinds, but he would be a bold man who would affirm that any of them gives complete satisfaction under all circumstances. The new douche submitted to us by the above firm is certainly in a high degree simple, portable, durable, and sterilisable. It is somewhat novel in form, which is of course no drawback, and it is efficient in use. The only respect in which, so far as can be seen, it may not prove perfectly convenient is that the instrument suitable for an adult would not, probably, be very manageable for a child; this could, of course, be overcome by manufacturing different sizes. The price is 25 cents.

